# Dopamine agonist serum concentrations and impulse control disorders in Parkinson's disease

**DOI:** 10.1111/ene.16144

**Published:** 2023-11-13

**Authors:** Sara C. Staubo, Ole Martin Fuskevåg, Mathias Toft, Ingeborg H. Lie, Kirsti M. J. Alvik, Pål Jostad, Stein H. Tingvoll, Hallvard Lilleng, Kristina Rosqvist, Elisabet Størset, Per Odin, Espen Dietrichs, Erik Sveberg Dietrichs

**Affiliations:** ^1^ Department of Neurology Oslo University Hospital Oslo Norway; ^2^ Department of Neurology Akershus University Hospital Nordbyhagen Norway; ^3^ Experimental and Clinical Pharmacology, Institute of Medical Biology UiT The Arctic University of Norway Tromsø Norway; ^4^ Department of Laboratory Medicine, Division of Diagnostic Services University Hospital of Northern Norway Tromsø Norway; ^5^ Department of Clinical Medicine UiT The Arctic University of Norway Tromsø Norway; ^6^ Institute of Clinical Medicine University of Oslo Oslo Norway; ^7^ Unicare Fram Rehabilitation Centre Rykkinn Norway; ^8^ Ringen Rehabilitation Centre Moelv Norway; ^9^ Department of Neurology University Hospital of Northern Norway Tromsø Norway; ^10^ Division of Neurology, Department of Clinical Sciences Lund University, Skåne University Hospital Lund Sweden; ^11^ Center for Psychopharmacology Diakonhjemmet Hospital Oslo Norway; ^12^ Institute of Oral Biology University of Oslo Oslo Norway

**Keywords:** dopamine agonist, impulse control disorders – pharmacology, Parkinson's disease

## Abstract

**Background and purpose:**

Impulse control disorders (ICDs) are common among Parkinson's disease patients using dopamine agonists. We wanted to determine whether ICD patients have higher dopamine agonist serum concentrations than those without any sign of ICD.

**Methods:**

Patients who used either pramipexole or ropinirole depot once daily were screened for ICDs using the validated Questionnaire for Impulsive‐Compulsive Disorders in Parkinson's Disease–Rating Scale. Those who scored above the cut‐off for one or more of the four defined ICDs (gambling, compulsive sexual behavior, compulsive shopping, and binge‐eating) were compared in a case–control study to patients who scored zero points (no evidence of ICD) on the same items. They were examined clinically and evaluated using relevant scales. Three blood samples were taken on the same day: before daily dose, and then 6 and 12 h later.

**Results:**

Forty‐six patients were included: 19 ICD‐positive and 27 controls. Ropinirole serum concentrations 6 h after daily intake (*C*_max_) were higher in the case group compared to the control group, as was the daily ropinirole dosage. No differences were observed in serum concentrations, dosage or total drug exposure for pramipexole. Disease duration and length of dopamine agonist treatment was significantly longer among ICD patients for ropinirole, but not for pramipexole.

**Conclusions:**

The use of pramipexole may in itself confer high ICD risk, whereas ICDs among ropinirole users depend more on serum concentration and drug exposure. The pharmacokinetic properties of ropinirole make it challenging to predict its effects on patients, which supports the need for therapeutic drug monitoring to reduce risk of ICD.

## INTRODUCTION

Motor symptoms in Parkinson's disease (PD) are mainly caused by degeneration of dopaminergic cells in the substantia nigra, with subsequent loss of dopaminergic neurotransmission in the striatum. Various medical treatments are available to alleviate Parkinsonian motor symptoms. Dopaminergic preparations are most effective and play a central role in modern Parkinson therapies, as evident from international evidence‐based guidelines [1, 2, 3, 4]. Most of these guidelines list both dopamine agonists and levodopa as possible first‐choice drugs, but there is an increasing concern about adverse effects of dopamine agonists, especially impulse control disorders (ICDs) [5, 6, 7, 8, 9, 10, 11, 12].

It has been known for many years that dopamine replacement therapy in PD may cause ICDs such as hypersexuality and pathological gambling [13, 14, 15, 16], but more recent studies have shown that ICDs are most common in patients using dopamine agonists [5, 6, 7, 8, 9, 10, 11, 12, 17, 18, 19]. Gambling, compulsive sexual behavior, compulsive shopping and binge‐eating are defined as ICDs, but many patients also experience other symptoms related to reduced impulse control, such as punding, hobbyism and dopamine dysregulation syndrome [20]. The incidence of ICDs in PD patients treated with dopamine agonists varies considerably among studies. In a cross‐sectional study of 3090 patients, Weintraub et al. [9] identified ICD in 17.1% of patients taking dopamine agonists. In PD patients not taking dopamine agonists, only 6.9% were classified with ICD. A multicenter longitudinal cohort study found a 51.5% 5‐year cumulative incidence of ICDs in patients who had ever taken dopamine agonists, whereas the incidence was 12.4% in patients who had never taken dopamine agonists [21]. Variable prevalence and incidence could be related to the various methods used to examine ICDs and, in some studies, to the inclusion of punding and other impulse control problems in addition to the four defined ICDs. Many reports are based on different research questionnaires [9, 10, 22, 23], while others are based on clinical interviews [7, 21, 24, 25].

The risk for developing ICDs seems to be highest among patients who use pramipexole or ropinirole [10]. Some of the published studies have reported that the occurrence of ICD is correlated with dopamine agonist dosage [21, 22, 24, 26]. This could imply that the risk of ICD development is linked to drug exposure, but dosage gives a poor estimate for the amount of active substance that reaches the target molecules and exerts pharmacological action in the patient. Ropinirole has a bioavailability ranging from 36% to 57%, meaning that the fraction of the administered dose that reaches the circulation varies largely among patients taking the same dose. Further, ropinirole elimination largely depends on liver‐mediated CYP1A2 metabolism, which is induced by smoking, for example, and could be inhibited by poor liver function, presumably having a substantial effect on ropinirole serum concentrations [27]. Pramipexole has a larger (90%) and more predictable bioavailability than ropinirole but is eliminated through the kidney, and serum concentrations would increase with kidney failure [28]. Thus, to estimate the dopamine agonist exposure in individual PD patients, therapeutic drug monitoring (TDM), measuring serum concentrations, would be far superior to dose assessment.

Possible relationships among ICD development, dopamine agonist dose and serum concentrations have so far not been documented. Only one previous study has examined ICD prevalence and dopamine agonist levels in plasma or serum [29]. The authors of that study found that plasma levels, when measured at the assumed minimal concentration (*C*_min_) shortly before the next dopamine agonist dose, were similar in patients with and without ICD.

We wanted to explore further the possible relationship between dopamine agonist medication levels and ICDs in PD patients by performing a detailed pharmacological (TDM) study. By assessing both trough levels (*C*_min_), *C*_max_ and total drug exposure, measured as area under the curve (AUC), of ropinirole and pramipexole in treated patients, we obtained detailed pharmacological information in patients with and without ICD.

## MATERIALS AND METHODS

The ICD Parkinson Agonist Pharmacology Study (IPAPS) was a cross‐sectional observational multicenter study. A total of 100 patients were screened during the period from spring 2020 to fall 2022 from Oslo University Hospital, University Hospital of Northern Norway, Ringen Rehabilitation Center and Unicare Fram Rehabilitation Center (all in Norway), and Skåne University Hospital Lund (Sweden). Patients were eligible if they had a diagnosis of idiopathic PD according to the International Parkinson and Movement Disorder Society (MDS) clinical diagnostic criteria [30] that was confirmed by a movement disorder specialist, and used either pramipexole or ropinirole depot once daily in the morning. No change of dopaminergic medication during the last month was allowed. Only non‐demented patients were eligible. No formal cognitive assessments were performed, but all patients went through extensive clinical interviews and only patients with no sign of cognitive impairment were invited to participate. Participants had to be available for clinical examination and three blood tests during a 12‐h period and had to be able to fill in all study forms. Patients who were willing to participate and signed the informed consent form were included whether they had experienced ICD symptoms or not. Other antiparkinsonian therapies were allowed. None of our patients used apomorphine (injection or pump treatment) or levodopa intestinal gel, two patients had undergone bilateral subthalamic nucleus deep brain stimulation (STN‐DBS).

Each patient went through neurological examination including a careful examination of motor function. This included scoring of the MDS‐Unified Parkinson's Disease Rating Scale (MDS‐UPDRS) parts III (ON medication) and IV [31], the Hoehn and Yahr rating scale [32], and classification of PD by the examiner (tremor dominant/rigid‐akinetic/mixed). The patients were interviewed about PD symptom debut, time of diagnosis, treatment history, comorbidities and ICD‐related problems. They completed the validated Questionnaire for Impulsive‐Compulsive Disorders in Parkinson's Disease–Rating Scale (QUIP‐RS) [33], the Non‐Motor Symptoms Questionnaire (NMSQ) [34], and the Parkinson's Disease Questionnaire (PDQ‐39) for health‐related quality of life [35].

### Case–control study

In this case–control study we compared patients with ICD to controls without any evidence of impulse control problems. Twenty of the 100 patients who underwent screening scored above the cut‐off values for at least one ICD (items A: gambling, B: hypersexuality, C: shopping, D: eating) on the QUIP‐RS [33]. Each of these items are scored from 0 to 16 points. Validated cut‐off values are ≥6 for gambling, ≥8 for hypersexuality, ≥8 for shopping, and ≥7 for eating [33]. Twenty‐eight of the 100 patients scored zero points on all these four items and served as controls. One ICD‐positive patient and one control were omitted due to incomplete pharmacological data. A total of 19 ICD‐positive patients and 27 controls were thus included. The patients were compared regarding dopamine agonist serum concentrations, current and previous medication, demographic data, disease history, clinical presentation, and scores for the MDS‐UPDRS, Hoehn and Yahr scale, NMSQ, and PDQ‐39.

### Blood tests and pharmacological analyses

Three blood tests were taken from each participant on the same day. The first sample was taken in the morning, immediately prior to the normal daily dose of dopamine agonist, at the assumed minimal serum concentration (*C*_min_). The second sample was collected after 6 h at the assumed maximal serum concentration (*C*_max_), on the basis that both ropinirole and pramipexole depot formulations reach *C*_max_ at approximately 6 h after intake. A third sample was collected after 12 h to enable calculation of the AUC from 0 to 24 h (AUC_0–24 h_). After sampling, blood was centrifuged and the serum was extracted and immediately frozen. Frozen blood samples were sent to the University Hospital in Northern Norway for analyses.

For measurements of ropinirole [36, 37, 38, 39] and pramipexole [38, 40] in serum, we used a validated method using liquid chromatography connected to a tandem mass spectrometer (LC–MS/MS). UniSpray was used for ionization. Preparation of samples was based on liquid–liquid extraction of analytes as well as isotope‐labeled ropinirole and pramipexole as internal standards to minimize matrix effects. Details of the analyses are given in Appendix S1.

### Area under the curve calculation and statistical analyses

For each participant the AUC_0–24 h_ was calculated using non‐compartmental analysis based on samples at 0 h (*C*_min_), 6 h and 12 h. The predose sample at 0 h was assumed to be the same as at 24 h (not measured). As no change of dopaminergic medication during the last month before inclusion was allowed, we assumed steady‐state pharmacokinetics. AUC_0–24 h_ calculations were performed using the linear up/log down trapezoidal method with the package “PK” in R v.4.2.1.

All reported values are presented as mean ± standard deviation. For both the ropinirole and pramipexole groups, case values were compared to control values using Sigma Plot 14.5 software. Due to limited sample size of the included groups, differences were statistically tested using a non‐parametric Mann–Whitney rank sum test.

### Ethical considerations

The study was approved by the Personvernombudet/Datatilsynet (General Data Protection Regulation in Norway; reference: 2018/6255), the Regional Ethical Committee in Northern Norway (reference: 2018/1343/REK nord), and the Swedish Ethical Board (reference: 2022‐01340‐01). Written informed consent was obtained from each study participant before inclusion.

## RESULTS

Forty‐six patients were included in this case–control study, 27 of whom served as controls (Table 1). Out of 19 ICD‐positive patients, seven had scores above the validated cut‐off values for two ICDs (items A–D on the QUIP‐RS), while the rest scored above cut‐off for one ICD. Three were positive for gambling (one female, two male), nine for hypersexuality (one female, eight male), four for shopping (two female, two male) and 10 for eating (seven female, three male). During the interview, all patients were asked if they personally felt ICD to be a problem. Two ICD‐positive patients reported ICD “as a serious problem”, two reported it as “bothersome”, eight as “to some extent”, and seven as “not at all”. All patients in the control group replied, “not at all”.

**TABLE 1:** Demographic data for ropinirole and pramipexole patients with and without impulse control disorder.

	Control	Case
	QUIP‐RS A–D score 0	QUIP‐RS A–D score > cut‐off
Total number	27	19
Number of patients using pramipexole	10	9
Number of patients using ropinirole	17	10
Gender	14 Male; 13 Female	12 Male; 7 Female
Mean (range) age, years	62 (46–78)	62 (42–87)

Abbreviation: QUIP‐RS, Questionnaire for Impulsive‐Compulsive Disorders in Parkinson's Disease–Rating Scale.

Of the 19 ICD‐positive patients, four (21%) were classified as having tremor‐dominant, 10 (53%) akinetic‐rigid, and five mixed‐type PD. In the control group, 10 of the 27 (37%) had tremor‐dominant PD, 12 (44%) akinetic‐rigid, and five mixed‐type.

### Pharmacology

**FIGURE 1:**
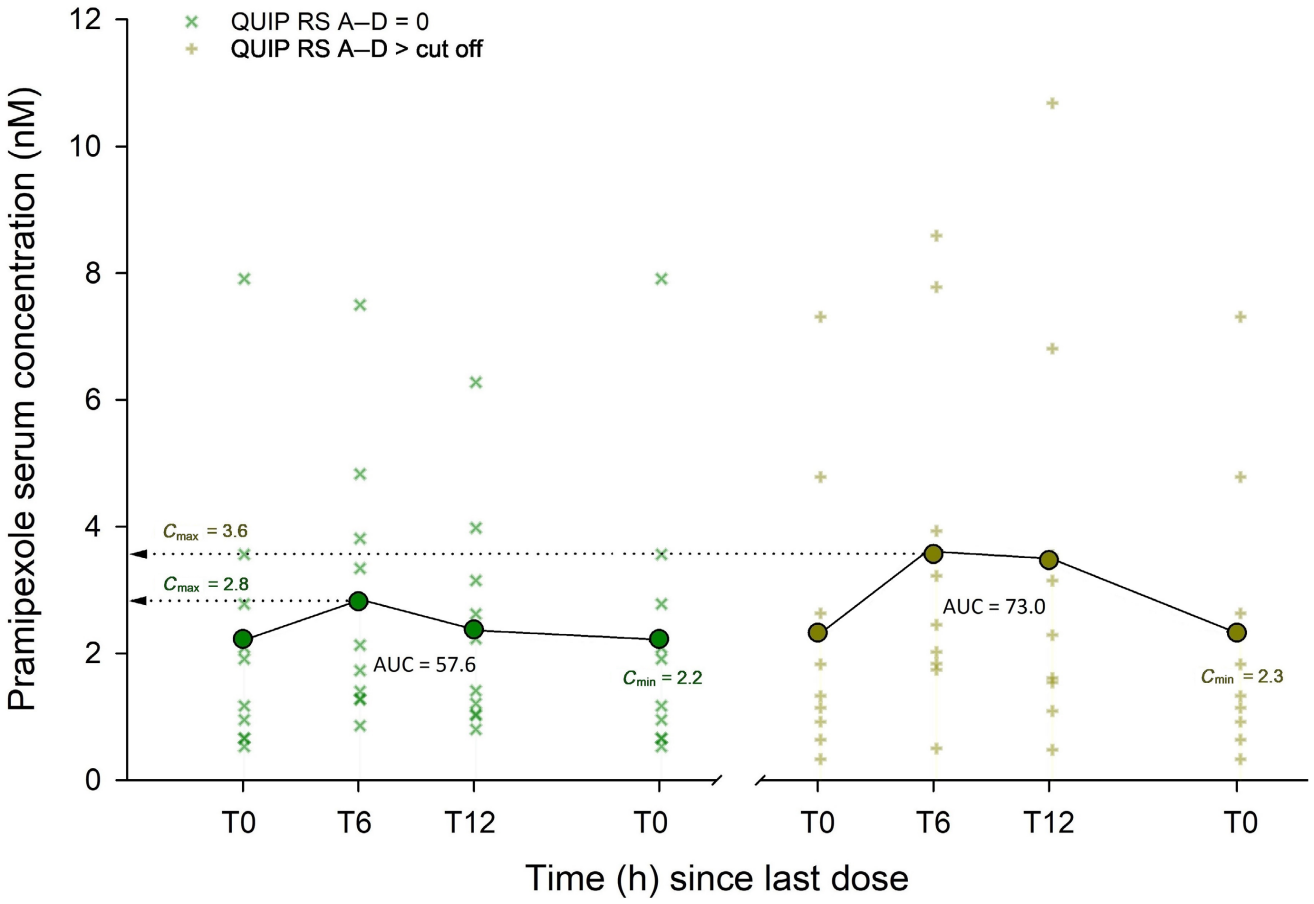
Diagrams showing pramipexole serum concentrations just before daily dose intake (T0), and after 6 h and 12 h. The control group is shown to the left, impulse control disorder (ICD)‐positive patients to the right. Area under the curve (AUC_0–24 h_) was calculated assuming T24 = T0. IPAPS, ICD Parkinson Agonist Pharmacology Study; QUIP‐RS, Questionnaire for Impulsive‐Compulsive Disorders in Parkinson's Disease–Rating Scale.

**FIGURE 2:**
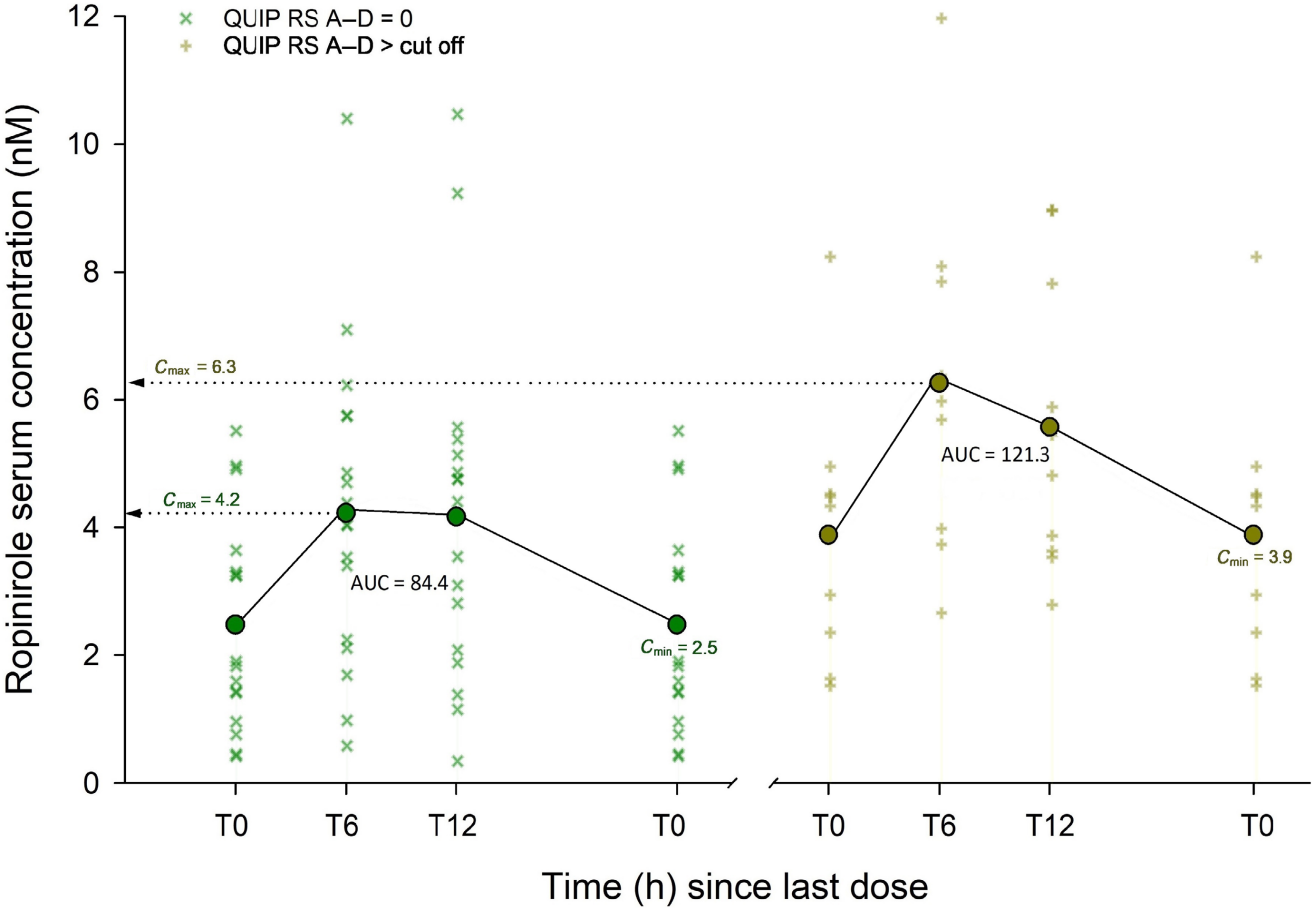
Diagrams showing ropinirole serum concentrations just before daily dose intake (T0), and after 6 h and 12 h. The control group is shown to the left, impulse control disorder (ICD)‐positive patients to the right. Area under the curve (AUC_0–24 h_) was calculated assuming T24 = T0. IPAPS, ICD Parkinson Agonist Pharmacology Study; QUIP‐RS, Questionnaire for Impulsive‐Compulsive Disorders in Parkinson's Disease–Rating Scale.

The serum concentrations 6 h after the daily intake of medication was higher in the case group compared to the control group for ropinirole (Table 2, Figures 1 and 2). In addition, the ropinirole dosage was significantly higher in the case group. The total drug exposure for ropinirole (AUC_0–24 h_) was higher in the case group but did not reach significance. The ratio between drug serum concentration at T0 and dosage (C/D ratio) for all ropinirole patients (case and control) was 0.455 ± 0.550 (coefficient of variation [CV]: 121%), and 1.35 ± 0.761 (CV: 56%) for pramipexole. There were no differences in serum concentrations or AUC_0–24 h_ between the two groups for pramipexole.

**TABLE 2:** Pharmacokinetic data for ropinirole and pramipexole patients with and without impulse control disorder.

Sample	Ropinirole			Pramipexole		
	Control	Case	*p* value	Control	Case	*p* value
	QUIP‐RS A–D = 0	QUIP‐RS A–D > cut‐off		QUIP‐RS A–D = 0	QUIP‐RS A–D > cut‐off	
Dosage, mg	8.4 ± 4.9	12 ± 2.8	0.05	1.6 ± 1.1	1.5 ± 0.6	0.84
Serum concentration 0 h/*C*_min_, nM	2.5 ± 1.6	3.9 ± 2.0	0.08	2.2 ± 2.2	2.3 ± 2.3	0.87
Serum concentration 6 h/*C*_max_, nM	4.2 ± 2.4	6.3 ± 2.7	0.05	2.8 ± 2.1	3.6 ± 2.8	0.41
Serum concentration 12 h, nM	4.2 ± 2.7	5.6 ± 2.3	0.16	2.4 ± 1.7	3.5 ± 3.3	0.44
AUC_0–24 h_, nM·h	84 ± 51	121 ± 50	0.08	58 ± 42	73 ± 67	0.62
Creatinine, μM	70 ± 17	67 ± 12	0.87	71 ± 9.4	88 ± 23	0.06
eGFR, ml/min/1.73m^2^	94 ± 13	101 ± 4	0.34	93 ± 11	83 ± 23	0.43
Age, years	63 ± 7.5	61 ± 7.4	0.45	59 ± 9.0	62 ± 14	0.74
Weight, kg	86 ± 21	78 ± 4.9	0.19	79 ± 9.0	86 ± 12	0.23

*Note*: Differences are compared statistically using a non‐parametric Mann–Whitney rank sum test. Values are given as mean ± standard deviation.

Abbreviations: AUC, area under the curve; eGFR, estimated GFR calculated using the CKD‐EPI formula; QUIP‐RS, Questionnaire for Impulsive‐Compulsive Disorders in Parkinson's Disease–Rating Scale.

### Disease characteristics

The duration of dopaminergic treatment was higher in the case group compared to the control group for both drugs (Table 3). The number of months with dopamine agonist treatment was higher in the case group compared to controls for ropinirole, but not for pramipexole.

**TABLE 3:** Disease and treatment characteristics of ropinirole and pramipexole patients in the case and control groups.

Sample	Ropinirole			Pramipexole		
	Control	Case	*p* value	Control	Case	*p* value
	QUIP‐RS A–D = 0	QUIP‐RS A–D > cut‐off		QUIP‐RS A–D = 0	QUIP‐RS A–D > cut‐off	
Months since symptom onset	97 ± 54	169 ± 73	0.02	75 ± 31	133 ± 65	0.06
Months since diagnosis	66 ± 49	145 ± 74	0.01	59 ± 33	107 ± 61	0.07
Months’ dopaminergic treatment	52 ± 43	140 ± 76	<0.01	41 ± 29	102 ± 59	0.05
Months’ ropinirole/pramipexole treatment	43 ± 42	110 ± 60	<0.01	52 ± 31	89 ± 61	0.16
Hoehn and Yahr score	2.2 ± 0.6	2.4 ± 0.3	0.32	1.6 ± 0.5	1.8 ± 0.7	0.36
Total LEDD score	670 ± 397	987 ± 320	0.06	752 ± 249	793 ± 372	0.81
MDS‐UPDRS III score	19 ± 9.1	27 ± 17	0.21	11 ± 5.3	19 ± 9.8	0.04
MDS‐UPDRS IV score	1.7 ± 2.0	6.2 ± 5.2	<0.01	0.5 ± 1.0	3.1 ± 2.9	0.03
NMSQ score	7.9 ± 4.8	13 ± 4.8	0.02	5.3 ± 2.7	11 ± 3.5	<0.01
PDQ‐39 SI score	32 ± 10	47 ± 13	<0.01	27 ± 6.7	42 ± 9.4	<0.01

*Note*: Differences are compared statistically using a non‐parametric Mann–Whitney rank sum test. Values are given as mean ± standard deviation.

Abbreviations: LEDD, levodopa equivalent daily dose; MDS‐UPDRS, International Parkinson and Movement Disorder Society‐Unified Parkinson's Disease Rating Scale; NMSQ, Non‐Motor Symptoms Questionnaire; PDQ‐39 SI, Parkinson's Disease Questionnaire Summary Index; QUIP‐RS, Questionnaire for Impulsive‐Compulsive Disorders in Parkinson's Disease–Rating Scale.

The ropinirole case group had longer disease duration than the control group. This was not found for pramipexole. The NMSQ and PDQ scores were higher (indicating worse symptoms) in the case group compared to the control group for both drugs. It appears from all rows in Table 3 that ropinirole ICD‐positive patients were more severely affected than their pramipexole counterparts: Longer disease duration, longer dopaminergic treatment, higher Hoehn and Yahr, MDS‐UPDRS III and IV, NMSQ and PDQ‐39 scores, and higher total levodopa equivalent daily dose (LEDD).

Table 4 shows other relevant scores. For ropinirole, MDS‐UPDRS IV scores from both items 4.1–4.2 (dyskinesias) and 4.3–4.6 (motor fluctuations) were significantly higher in the case group compared to the control group. For pramipexole, significant results were found only for motor fluctuations.

**TABLE 4:** Subscores from the International Parkinson and Movement Disorder Society‐Unified Parkinson's Disease Rating Scale and Parkinson's Disease Questionnaire‐39

Sample	Ropinirole			Pramipexole		
	Control	Case	*p* value	Control	Case	*p* value
	QUIP‐RS A–D = 0	QUIP‐RS A–D > cut‐off		QUIP‐RS A–D = 0	QUIP‐RS A–D > cut‐off	
MDS‐UPDRS III 3.1–3.14	15 ± 6.7	24 ± 13	0.06	8.0 ± 4.8	16 ± 11	0.06
MDS‐UPDRS III 3.15–3.18	3.7 ± 4.5	3.7 ± 4.1	0.84	2.6 ± 2.7	2.9 ± 3.0	0.80
MDS‐UPDRS IV 4.1–4.2	0.41 ± 0.80	1.9 ± 1.9	0.01	0.0 ± 0.0	0.67 ± 1.4	0.13
MDS‐UPDRS IV 4.3–4.6	1.2 ± 1.6	4.3 ± 3.8	0.02	0.50 ± 0.97	2.4 ± 2.3	0.04
PDQ‐39 q1–10	34 ± 16	46 ± 16	0.02	23 ± 3.9	40 ± 13	<0.01
PDQ‐39 q11–16	34 ± 12	51 ± 19	0.02	25 ± 9.0	46 ± 10	<0.01
PDQ‐39 q17–22	32 ± 15	49 ± 18	0.01	29 ± 10	42 ± 13	0.02
PDQ‐39 23–26	29 ± 13	52 ± 18	<0.01	26 ± 8.6	37 ± 17	0.10
PDQ‐39 q27–29	26 ± 14	47 ± 21	<0.01	25 ± 10	44 ± 23	0.18
PDQ‐39 q30–33	35 ± 12	41 ± 15	0.36	34 ± 14	40 ± 12	0.01
PDQ‐39 q34–36	28 ± 12	41 ± 16	0.03	25 ± 13	40 ± 21	0.02
PDQ‐39 q37–39	38 ± 14	51 ± 26	0.17	31 ± 14	45 ± 13	0.04

*Note*: Values are given as mean ± standard deviation. Differences are compared statistically using a non‐parametric Mann–Whitney rank sum test.

Abbreviations: MDS‐UPDRS, International Parkinson and Movement Disorder Society‐Unified Parkinson's Disease Rating Scale; PDQ‐39, Parkinson's Disease Questionnaire; QUIP‐RS, Questionnaire for Impulsive‐Compulsive Disorders in Parkinson's Disease–Rating Scale.

## DISCUSSION

In this pharmacological case–control study of PD patients using pramipexole or ropinirole, we compared those scoring above cut‐off for one or more ICDs to those scoring zero points (no evidence of ICD) for the same items on the validated QUIP‐RS form. The main finding was that ICD‐positive ropinirole users had higher serum concentrations at assumed *C*_max_, 6 h after their daily dopamine agonist intake, compared to controls without any evidence of ICD. Mean AUC_0–24 h_ representing total ropinirole exposure also appeared higher in the ICD‐positive group. Similar observations were not seen among pramipexole users. Only one pharmacological study has previously addressed the association between dopamine agonist serum concentrations and ICDs, but they found that plasma levels at *C*_min_ were similar between patients with and without ICD [29]. A direct association between agonist doses and ICD risk has previously been reported by several authors, although not describing differences between the different dopamine agonists [21, 22, 24, 26].

We found a higher ropinirole dose among ICD‐positive patients compared to controls. There were no dose differences between ICD‐positive and ICD‐negative pramipexole users. More unpredictable serum concentrations after administering ropinirole (C/D ratio CV: 121%) than pramipexole (C/D‐ratio CV: 56%), owing to differences in bioavailability and elimination, might be one explanation. These pharmacokinetic properties would make it more difficult for the treating clinician to predict dose‐dependent drug exposure in a ropinirole‐treated patient than in a patient receiving pramipexole, as we show in the present study. Low to normal ropinirole doses could give high serum concentrations and increased risk of ICD in a few patients, while the majority would tolerate it well. For pramipexole there is a clearer dose–concentration relationship.

Furthermore, our observations implicate differences between the pharmacodynamic properties of pramipexole and ropinirole, where the use of pramipexole in itself may confer high risk for ICDs, whereas ICD risk among ropinirole users is more dependent on drug exposure. Time to onset of ICD and test scores in pramipexole and ropinirole patients in our data could support this. ICD‐positive ropinirole patients had higher mean values for disease duration, dopaminergic treatment, months’ use of the dopamine agonist, higher Hoehn and Yahr score, higher LEDD, higher MDS‐UPDRS III and IV scores, higher NMSQ score and higher PDQ‐39 SI score compared to the ICD‐positive pramipexole users (Table 3). This suggests that there is an increased risk for ropinirole users to develop ICD with elevated drug exposure. For pramipexole users, the same relationship does not seem to exist and might imply that exposure to clinically relevant doses of this drug is sufficient to trigger ICD onset, independent of bioavailability and drug elimination in predisposed individuals. The shorter mean duration of agonist treatment in the pramipexole case group than the ropinirole case group could support this hypothesis, as ICD in pramipexole patients seems more closely related to time of drug exposure than to pharmacokinetic properties.

The pathophysiological mechanisms causing ICD remain unclear, but dopaminergic treatment affecting the dopamine D2 and D3 receptors of the mesolimbic pathway seem to be implicated in ICD development [41]. Both pramipexole and ropinirole have high selectivity for both D2 and D3 receptors compared to other dopamine agonists [42], and these are the two agonists associated with the highest occurrence of ICDs [10]. However, individual dispositions seem to be important, and a growing body of data suggests that specific dopamine receptor genetic polymorphisms may be important risk factors for ICD development. Thus, genetic profiling with calculation of a dopamine genetic risk score (DGRS) from known polymorphisms in genes for D1, D2 and D3 receptors, as well as dopamine transporter and cathecol‐O‐methyltransferase, has been proposed as a means of identifying high‐risk patients [43, 44]. If this theory were applied to the use of pramipexole, it could even explain how long‐term exposure can be detrimental for impulse control although serum‐concentrations do not differ from non‐ICD patients, as seen in our study. For ropinirole patients however, serum concentrations (*C*_max_) did correlate with risk for ICD. One could speculate that TDM could be an even more powerful tool, after calculation of DGRS both in ropinirole and pramipexole patients. It is likely that patients with a high risk score would be more sensitive to high dopamine agonist serum concentrations. Accordingly, calculation of DGRS, together with TDM, could be useful tools to predict ICD risk over time.

Payer et al. [45] did not find an increased number of dopamine D3 receptors among PD patients with ICD. However, ICD does seem to be related to early development of PD and the rs6280 single nucleotide variant of the dopamine D3 receptor gene [46]. Further, increased presence of dyskinesias has been found in ICD patients [47], and Biundo et al. [48] found that more than half of all patients with dyskinesia and PD had ICD. In our analysis, a higher dyskinesia burden (MDS‐UPDRS IV items 4.1 and 4.2) was found for ropinirole, but not for pramipexole ICD‐positive patients.

Two out of the 100 patients who were screened for this case–control study were treated with STN‐DBS long before this study and without any evidence of behavioral changes after the start of DBS treatment. We chose to examine these two patients even though a tendency towards increased impulsivity has been reported after STN‐DBS [49]. Both these patients used pramipexole. One of them scored above cut‐off for one ICD and was included in this study.

An obvious weakness in this study is the low number of patients included. Small between‐group differences may have escaped recognition, both pharmacologically and clinically. Among the study's strengths is that the participants’ blood samples were tested at *C*_min_, immediately before their daily dose of dopamine agonist and after 6 and 12 h on the same day. This allowed us to measure serum concentration variations as well as calculate the total drug exposure (AUC_0–24 h_). This is in contrast to a recent study that reported only *C*_min_ values for dopamine agonists in ICD patients, which did not differ from the non‐ICD control group [29]. Similarly to the present study, however, that study showed a non‐significantly higher mean ropinirole plasma concentration in ICD patients, while mean concentrations of pramipexole were almost equal in the two groups. Furthermore, in the present study, we showed that ropinirole ICD patients had *C*_min_ values (3.9 nM) that were almost equal to the *C*_max_ values (4.2 nM) of the non‐ICD patients, who had a *C*_min_ concentration of 2.5 nM. This indicates that TDM could be used to reduce risk of ICD when administering ropinirole. A target *C*_min_ serum concentration between 2 and 3 nM could be used to reduce risk of ICD onset. When more effective symptomatic treatment is needed, other dopaminergic treatment options should be considered.

Our patients were clinically well characterized through interview, neurological examination and various assessment forms, with some data self‐reported and some scored by the attending neurologist. Only patients scoring above the QUIP‐RS cut‐off for at least one of the four defined ICDs and controls scoring zero points on the same items were compared. This was important because our personal experience is that many patients underreport their ICD problems. Seven of our ICD‐positive patients reported no problems related to impulse control during the clinical interview but scored above the validated cut‐offs on the QUIP‐RS form. When some of these patients were asked again, after completing the QUIP‐RS form, they confirmed the presence of ICD problems.

The present study was planned and conducted as a case–control study, and fewer than half of all examined patients were included. Further correlation analyses in which we include all patients, even those with sub‐threshold scores, are planned.

In conclusion, our study shows that ICD‐positive ropinirole patients have higher serum concentrations than controls at assumed *C*_max_ to 6 h after drug intake, and that ICD risk increases with ropinirole dose and disease progression. The same results were not observed for pramipexole. These findings may indicate that dose reduction could be a possible strategy for treating ICD problems in ropinirole patients and that a target *C*_min_ concentration of 2–3 nM could be used to reduce ICD risk in ropinirole patients, but that a similar strategy would be less effective for pramipexole.

## AUTHOR CONTRIBUTIONS

**Espen Dietrichs:** Conceptualization; methodology; data curation; investigation; formal analysis; supervision; funding acquisition; project administration; resources; writing – original draft; writing – review and editing. **Sara C. Staubo:** Conceptualization; writing – original draft; writing – review and editing; data curation; formal analysis; investigation; methodology. **Ole Martin Fuskevåg:** Data curation; formal analysis; methodology; writing – review and editing. **Mathias Toft:** Data curation; investigation; resources; writing – review and editing. **Ingeborg H. Lie:** Data curation; investigation; writing – review and editing. **Kirsti M. J. Alvik:** Data curation; investigation; resources; writing – review and editing. **Pål Jostad:** Investigation; data curation; resources; writing – review and editing. **Stein H. Tingvoll:** Data curation; investigation; resources; writing – review and editing. **Hallvard Lilleng:** Investigation; data curation; resources; writing – review and editing. **Kristina Rosqvist:** Data curation; investigation; resources; writing – review and editing. **Elisabet Størset:** Methodology; data curation; writing – review and editing; formal analysis. **Per Odin:** Data curation; writing – review and editing; investigation; resources. **Erik Sveberg Dietrichs:** Conceptualization; methodology; data curation; investigation; formal analysis; supervision; funding acquisition; project administration; resources; writing – original draft; writing – review and editing.

## CONFLICT OF INTEREST STATEMENT

The authors declare that there are no conflicts of interest relevant to this work. Per Odin has given lectures and/or been advisor with honorarium for AbbVie, Bial, Britannia, Global Kinetics, Lobsor, Lundbeck, Nordic Infucare, Stada, UCB and Zambon, and has participated as an investigator in clinical studies performed by AbbVie, Global Kinetics, IRL, UCB and Zambon. Espen Dietrichs has given lectures and/or been advisor with honorarium for AbbVie, Global Kinetics, Ipsen and Nordic Infucare. The other authors have nothing to declare.

## Supporting information


Appendix S1

## Data Availability

The data that support the findings of this study are available from the corresponding author upon reasonable request.
