# Supplementary material for: Dopamine agonist serum concentrations and impulse control disorders in Parkinson's disease
Source: Eur J Neurol. 2023 Nov 13;31(2):e16144. doi: 10.1111/ene.16144 (PMC11235607; doi:10.1111/ene.16144)
Supplement: Supplementary file 1 — Appendix S1 [file ENE-31-e16144-s001.docx]

**IPAPS Analyses - Supplementary Information**

**Chemicals and reagents**

Pramipexole, ropinirole, pramipexole-d4 and ropinirole-d3 were purchased from TRC (Toronto Research Chemicals, Ontario, Canada). LC-MS grade methanol was obtained from Honeywell*^™^* Riedel-de Häen^™^ (Seelze, Germany). LC-MS grade ammonium acetate was purchased from Sigma-Aldrich (Steinheim,Germany*).* Analytical grade ethyl acetate and LC-MS grade formic acid were supplied by Merck (Darmstadt, Germany) and tert-butyl methyl ether was obtained from Supelco (Sigma-Aldrich, Steinheim,Germany). Ultrapure water (18.2 MΩ) was obtained from a Millipore Integral 5 system (Millipore SAS, Molsheim, France).

**Preparation of standards**

Stock solutions of ropinirole and pramipexole (1793 and 636 µM, respectively) were prepared in LC-MS grade methanol and stored at -35 °C. A 7-point calibration curve was prepared in technical serum by dilution of the stock solution at the following concentrations: 100, 50, 10, 5, 1, 0.5 and 0.1 nM. The samples, standard solutions and blank serum samples were stored at -80 °C until analysis. An internal standard solution was prepared by adding ropinirole-d4 and pramipexole-d3 stock solutions to ultrapure water to a final concentration of 10 nM.

**Liquid-Liquid extraction**

A Tecan Freedom Evo 200 (Männedorf, Switzerland) liquid handling workstation was used for sample preparation. Sample preparation procedure for samples was as follows: 80 µl of serum samples and standards were transferred to a 96-well MegaBlock^®^ before 50 µl aqueous internal standard and 500 µl tert-butyl methyl ether : ethyl acetate (1:1) was added to each wells. The plate was mixed on a Bioshake (QInstruments, Germany) at 1500 rpm for 3 min, sealed and centrifuged at 400 g for 5 min. at 15 °C in a Universal 320R centrifuge (Hettich Zentrifugen, Tuttlingen, Germany). A 350 μL aliquot of supernatant from the mixtures were transferred to a 96-well 1 ml collection plate (Waters, Milford, MA) and evaporated under a gentle stream of nitrogen at 40 °C using a MiniVap microplate evaporator (Porvair Sciences,Wales, UK). Sample residues were reconstituted in 50 μL of 0.1% formic acid with 2 mM ammonium acetate in water : methanol (1:1).

**Liquid chromatography tandem mass spectrometry**

Samples were analysed by LC-MS/MS using Waters Acquity^TM^ *I-*class UPLC system with an autosampler and a binary solvent delivery system (Waters, Milford, MA) interfaced to Waters Xevo TQ-XS benchtop tandem quadrupole mass spectrometer (Waters, Manchester, UK). The mass spectrometer was operated in positive UniSPray^®^ ion mode (US+) and impactor voltage was set to 0.5 kV. The system was controlled by the MassLynx version 4.2 software. Desolvation gas temperature was 500 °C; source temperature was 150 °C; desolvation gas flow was 1000 L/h; cone gas flow was 150 L/h; collision gas pressure was 4 × 10^-6^ Bar (argon) and the ion energies were 0.5 V for both quadrupoles. Separation was performed on an Acquity UPLC^®^ Cortecs T3, (1.6 µm, 2.1 mm i.d. × 100 mm, Waters, MA, USA). The injection volume was set to 1.0 µL. Eluent A consisted of 2 mM ammonium acetate in water; eluent B consisted of 0.1% formic acid in methanol. Gradient elution was performed with 5% B at start and had a linear increase to 40% B until 3 min, a linear increase to 99 % B until 3.5 min, hold at 99% B until 4.2 min, and re-equilibration until 6 min with 5% B. The flow rate was 0.3 mL/min and the column temperature was maintained at 50 °C. For quantitative analysis, the following multiple reaction monitoring (MRM) transitions for ropinirole and pramipexole and their deuterated analogues were used (bold transitions are qualifiers): *m/z* 261→160/**114** and 265→160/118 (ropinirole and ropinirole-d4), *m/z* 212→153/**111** and 215→153/111 (pramipexole and pramipexole-d3).

**Precision and accuracy**

The method was validated and found to be linear from 0.1 to at least 100 nM with a squared correlation coefficient (*r*^2^) > 0.995 for ropinirole and pramipexole. Lower limit of quantifications (LLOQ) for ropinirole and pramipexole were found to be 0.01 and 0,05 nM respectively. Between-day coefficients of variation (CVs) for pramipexole and ropinirole were < 6% on three consecutive days. Intraday precision values were evaluated by assaying three samples (low, medium and high concentration) six times on the same day. The CVs for pramipexole and ropinirole were < 4% for all three levels. Accuracy for recovery tests was between 95% and 104% (4 levels, *n* = 3 for each).
